# Validation and comparison of the molecular classifications of pancreatic carcinomas

**DOI:** 10.1186/s12943-017-0739-z

**Published:** 2017-11-06

**Authors:** David J. Birnbaum, Pascal Finetti, Daniel Birnbaum, Emilie Mamessier, François Bertucci

**Affiliations:** 1 Département d’Oncologie Moléculaire, Centre de Recherche en Cancérologie de Marseille, Institut Paoli-Calmettes, INSERM UMR1068, CNRS UMR725, Aix-Marseille Université, Marseille, France; 2 Département de Chirurgie Générale et Viscérale, AP-HM, Marseille, France; 3 Faculté de Médecine, Aix-Marseille Université, Marseille, France; 4 Département d’Oncologie Médicale, Institut Paoli-Calmettes, 232, Bd Ste-Marguerite, 13009 Marseille, France

**Keywords:** Expression, Molecular subtypes, Pancreatic cancer, Prognosis, Survival

## Abstract

Four molecular classifications of pancreatic ductal adenocarcinoma (PDAC), biologically and clinically relevant and based on gene expression profiles, were established in the recent years, including the Collisson’s, Moffitt’s (“tumor” and “stroma” classifications), and Bailey’s classifications. The aim of this study was to validate the prognostic value of the Moffitt’s classifications and to compare the Collisson’s, Moffitt’s, and Bailey’s classifications in a large series of samples. We collected clinical and gene expression data of PDAC samples from 15 public data sets, resulting in a total of 846 primary cancer samples, including 601 with survival annotation. All samples were classified according to each of the four multigene classifiers. We confirmed the independent prognostic value of the Moffitt “tumor”, Moffitt “stroma”, and Bailey’s classifications, but not that of the Collisson’s classification. Despite a relatively low gene overlap, all classifications were associated with pathological grade, an important prognostic feature and reflect of intrinsic molecular characteristics of tumors. The concordance rate in term of “good-prognosis” *vs*. “poor-prognosis” prediction by classifiers was relatively high (from 73 to 86%) between the three “tumor” classifications based on tumor gene lists (Collisson, Moffitt “tumor”, Bailey), but low (from 50 to 60%) with the Moffitt’s stroma classification based on stroma genes. Multivariate analysis incorporating the four classifiers together retained as significant variables the Moffitt “stroma” and Bailey classifications, highlighting the complementarity of classifiers based on tumor epithelium (Bailey) and tumor stroma (Moffitt stroma). Our results reinforce the clinical validity of subtyping in PDAC, which should be regarded as a collection of separate diseases. Beside their clinical utility that remains to be demonstrated, the clinical interest of the subtypes, notably those from Bailey’s and Moffitt’s “stroma” classifiers that show independent prognostic value, will be reinforced by the identification of new biomarkers and/or therapeutic targets in each subtype for designing and testing novel specific targeted therapies.

**Electronic supplementary material:**

The online version of this article (10.1186/s12943-017-0739-z) contains supplementary material, which is available to authorized users.

Pancreatic ductal adenocarcinoma (PDAC) is one of the most aggressive human cancers [1]. Its incidence is rising [2] and the therapeutic advances have achieved only limited impact. As demonstrated for breast cancer [3], the identification of molecular subtypes allows a better definition of the clinical heterogeneity of cancers and the design of targeted therapeutic strategies. Recently, three studies have identified biologically and clinically relevant molecular PDAC subtypes based on gene expression profiles. In 2011, Collisson et al. defined three subtypes (“classical”, “quasi-mesenchymal”, “exocrine-like”) based on surgical microdissected epithelial tumor samples and associated with overall survival (OS) in multivariate analysis in 27 informative samples [4]. Moffitt et al. “separated” the stroma from the epithelial pancreatic tumor by virtual microdissection and identified two “stroma subtypes” (“normal” and “activated”) with different OS in a 108-patients series, and two “tumor-specific subtypes” (“classical” and “basal-like”) with different OS in a 125-patients series with subsequent validation in 96 patients [5]. Bailey et al. defined four subtypes (“squamous”, “pancreatic progenitor”, “immunogenic”, “aberrantly differentiated endocrine exocrine (ADEX)”), associated with different OS in multivariate analysis in the series of 96 patients [6].

Two important questions remain regarding these classifications established in relatively small series. The first one concerns the robustness of their prognostic value, notably the Moffitt’s classification, the Collisson’s and the Bailey’s classifications having been recently challenged in respective series of 118 patients [7] and 364 patients ([8]. The second question is whether these classifications provide redundant clinical information regarding outcome prediction for individual patients. Here, we have analyzed a large series of 846 patients with two objectives: to validate the prognostic value of the Moffitt classifications and to compare the four classifications.

## Results and discussion

We collected clinical and gene expression data of PDAC samples from 15 public data sets (Additional file 1) selected according to the following criteria: availability of data in the GEO, Array-Express, EGA, or TCGA databases, and presence of at least 20 samples. Three data sets [4, 6, 9] had been previously used across the three earlier studies. A column indicating the sets previously used in these earlier studies The final set contained 846 primary cancer samples, including 819 non-microdissected samples and 27 Collisson’s epithelium microdissected samples (3% of cases). Before analysis, expression data were normalized as described [10]. Briefly, we first normalized each DNA microarray-based data set separately, by using quantile normalization for the available processed data from non-Affymetrix-based sets (Agilent, Illumina), and Robust Multichip Average (RMA) with the non-parametric quantile algorithm for the raw Affymetrix data sets. Then, we mapped hybridization probes across the different technological platforms present. We used SOURCE (http://smd.stanford.edu/cgi-bin/source/sourceSearch) and EntrezGene (*Homo sapiens* gene information db, release from 09/12/2008, ftp://ftp.ncbi.nlm.nih.gov/gene/) to retrieve and update the non-Affymetrix gene chips annotations, and NetAffx Annotation files (www.affymetrix.com; release from 01/12/2008) for the Affymetrix annotations. The probes were then mapped according to their EntrezGeneID. For the TCGA data, we used the available normalized RNASeq data that we log_2_-transformed. Finally, we defined the molecular subtype of each sample in each data set separately as defined in the original publications: the three Collisson’s subtypes [4], the two Moffitt’s “tumor-specific subtypes” [5], the two Moffitt’s “stroma subtypes” [5], and the four Bailey’s subtypes [6].

**Fig. 1:**
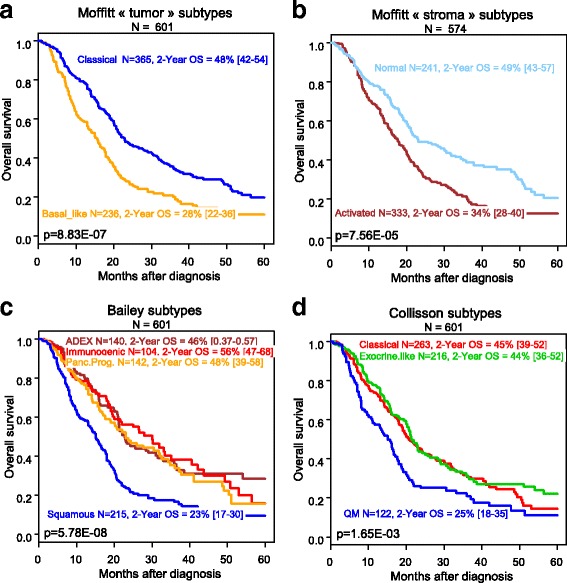
Overall survival in patients with pancreatic cancer according to the four molecular classifications. **a** Kaplan-Meier OS curves according to the two Moffitt’s “tumor” subtypes. **b** Similar to A/, but according to the two Moffitt’s “stroma” subtypes. **c** Similar to A/, but according to the four Bailey’s subtypes. **d** Similar to A/, but according to the three Collisson’s subtypes. *P*-value is for the log-rank test

We first searched for correlations between the Moffitt’s classifications (“tumor” and “stroma”) and clinical data (Additional file 2). Regarding the “tumor” classification, the “basal-like” subtype was associated with age ≤ 60 years, ductal type, pathological grade 3. Regarding the “stroma” classification, the “activated” subtype was associated with ductal type, grade 2, tumor size. Overall survival (OS), calculated from diagnosis to death, was available for 601 patients. With a median follow-up of 16 months, the 2-year OS was 40%. The 2-year OS rates for the Moffitt’s classifications were (Fig. 1a-b) 49% in the “classical” “tumor subtype” vs. 28% in the “basal-like” (*p* = 8.83E-07), and 49% in the “normal” “stroma subtype” vs. 34% in the “activated” (*p* = 7.56E-05). In univariate analysis (Table 1), the Moffitt’s classifications (“tumor”: *p* = 1.17E-06; “stroma”: *p* = 8.46E-05) were associated with OS, as were the AJJCC stage, the pathological type, grade, tumor size, lymph node status. In multivariate analysis (Table 1), each of them remained significant (“tumor”: *p* = 1.56E-03; “stroma”: *p* = 4.25E-02).

**Table 1:** Uni- and multivariate Cox regression analyses for OS including the four molecular classifications

Characteristics		Univariate			Multivariate** w/ Moffitt tumor			Multivariate** w/ Moffitt stroma			Multivariate** w/ Collisson			Multivariate** w/ Bailey			Multivariate** w/ four classifications		
		N	HR [95CI]	P*	N	HR[95CI]	P*	N	HR[95CI]	P*	N	HR[95CI]	P*	N	HR[95CI]	P*	N	HR[95CI]	P*
Age at diagnosis	>60 vs. ≤60 years	364	1.21 [0.89–1.65]	0.233															
Sex	male vs. female	367	1.13 [0.85–1.50]	0.392															
AJCC Stage	2 vs. 1	515	2.36 [1.56–3.58]	***2.16E-04***	215	1.52[0.24–9.63]	0.658	209	0.56[0.1–3.32]	0.527	215	0.64 [0.1–4.01]	0.632	215	0.97 [0.14–6.62]	0.979			
	3 vs. 1		3.54 [1.53–8.22]		215	24.42[1.46–408]	***2.61E-02***	209	<NA>[NA-NA]	<NA>	215	13.58[0.83–222.5]	0.067	215	19.9[1.15–345.4]	0.040			
	4 vs. 1		3.56 [1.53–8.30]		215	0.00[0.00-Inf]	0.999	209	0.00[0.00-Inf]	0.999	215	0 [0-Inf]	0.998	215	0 [0-Inf]	0.999			
Pathological type	other vs. ductal	548	0.14 [0.03–0.55]	***5.27E-03***	215	0.00[0.00-Inf]	0.996	209	0.00[0.00-Inf]	0.996	215	0 [0-Inf]	0.996	215	0 [0-Inf]	0.996			
Pathological grade	2 vs. 1	280	2.67 [1.15–6.21]	***3.14E-03***	215	1.34[0.55–3.25]	0.521	209	1.08[0.44–2.69]	0.862	215	1.39 [0.57–3.38]	0.470	215	1.31 [0.54–3.2]	0.556			
	3 vs. 1		4.19 [1.79–9.81]		215	1.71[0.7–4.18]	0.242	209	1.29[0.53–3.18]	0.574	215	1.55 [0.64–3.79]	0.332	215	1.47 [0.6–3.58]	0.400			
	4 vs. 1		4.78 [0.96–23.8]		215	3.25[0.36–29.4]	0.295	209	3.66[0.4–33.48]	0.251	215	6.62 [0.61–71.8]	0.120	215	3.27 [0.35–30.11]	0.296			
pT	2 vs. 1	393	1.81 [0.75–4.36]	***1.85E-02***	215	2.03[0.23–17.9]	0.524	209	2.28[0.26–20.18]	0.460	215	3.37 [0.37–30.38]	0.280	215	1.75 [0.19–16.17]	0.623			
	3 vs. 1		2.64 [1.16–5.98]		215	2.74[0.19–38.8]	0.456	209	4.61[0.33–63.47]	0.254	215	5.66 [0.39–81.41]	0.203	215	3.33 [0.22–51.1]	0.389			
	4 vs. 1		3.90 [1.35–11.2]		215	<NA>[NA-NA]	<NA>	209	<NA>[NA-NA]	<NA>	215	<NA> [NA-NA]	<NA>	215	<NA> [NA-NA]	<NA>			
pN	1 vs. 0	451	2.01 [1.5–2.7]	***2.86E-06***	215	1.37[0.78–2.39]	0.270	209	1.42[0.81–2.5]	0.219	215	1.62 [0.93–2.84]	0.090	215	1.4 [0.78–2.54]	0.261			
Moffitt “tumor”	classical vs. basal-like	601	0.59 [0.48–0.73]	***1.17E-06***	215	0.43[0.26–0.73]	***1.56E-03***										574	0.91[0.67–1.24]	0.557
Moffitt “stroma”	normal vs. activated	574	0.64 [0.51–0.8]	***8.46E-05***				209	0.56[0.32–0.98]	***4.25E-02***							574	0.73[0.57–0.92]	***9.07E-03***
Collisson	exocrine-like vs. classical	601	0.90 [0.71–1.15]	***1.79E-03***							215	0.74 [0.44–1.25]	0.266				574	0.96[0.68–1.35]	0.801
	quasi-mesenchymal vs. classical		1.47 [1.13–1.91]								215	0.75 [0.29–1.95]	0.561				574	0.92[0.66–1.28]	0.615
Bailey	immunogenic vs. ADEX	601	1.00 [0.70–1.42]	***1.03E-07***										215	0.98 [0.43–2.24]	0.961	574	0.93[0.59–1.47]	0.759
	pancreatic progenitor vs. ADEX		1.13 [0.82–1.57]											215	1.64 [0.78–3.45]	0.196	574	1.06[0.69–1.63]	0.777
	squamous vs. *ADEX		1.99 [1.50–2.65]											215	2.3 [1.16–4.55]	***1.69E-02***	574	1.69[1.09–2.62]	***1.94E-02***

*P-value for Wald test; **the multivariate analysis included all variables significant (*p* < =0,05) in univariate analysis; pT, pathological tumor size (pT); pN, pathological lymph node status. The *P*-values in boldface and italicized represent the significant *P*-values

**Fig. 2:**
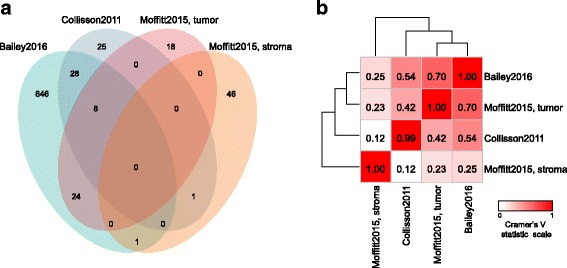
Comparison of the four molecular classifications. **a** Venn diagram comparing the gene lists of the four subtype classifications. **b** Heatmap of Cramer’s V statistic reflecting the strength of the correlations between the classifiers in term of assignment to the poor-prognosis and good-prognosis groups. The V statistic values are color-coded according to the scale shown below the heatmap

We then compared the four classifications according to several criteria. Regarding the gene composition, the crossing of the four gene lists (Bailey: 707 genes; Collisson: 62 genes; Moffitt’s “tumor”: 50 genes; Moffitt’s “stroma”: 48 genes) showed (Fig. 2a **,** Additional file 3) many more genes in common between the Bailey’s, Collisson’s, and Moffitt’s “tumor” lists - respectively derived from bulk tumor tissues, microdissected tumor tissues, and bulk tumor tissues but with virtual microdissection retaining the tumor epithelial cell genes - than between each of them and the Moffitt’s “stroma” list, derived from tumor tissues but with virtual microdissection retaining the stromal genes only. Thirty-seven of 62 Collisson’s genes (58%) and 32 of 50 Moffitt’s “tumor” genes (64%) were included in the Bailey’s list in which they represented only 5% of genes, whereas 8 of 62 Collisson’s genes (13%) were included in the Moffitt’s “tumor” list, in which they represented 16% of genes. There was only one gene in common between the Bailey’s or Collisson’s lists and the Moffitt’s “stroma” list (Additional file 4). The mean percentage of common genes between each list and the three other ones was 3% for the Bailey’s list, 24% for the Collisson’s list, 27% for the Moffitt “tumor” list, and 1% for the Moffitt “stroma” list, suggesting little overlap. Several methodological explanations account at least in part for this discrepancy, as reported for prognostic signatures in breast cancer [11, 12]: different samples (whole-tumor for Bailey, microdissection for Collisson, and virtual microdissection for Moffitt), different patients, different technological platforms (DNA microarrays, RNA-Seq) with different tested gene sets for DNA microarrays, different methods of data handling, notably different cut-offs of significance for the retained genes, But the discordance may also be only apparent because discriminator genes, even if different through classifiers, may be involved in the same pathways or cell processes.

We compared the correlations between all classifications and clinicopathological data **(**Additional file 2**)**. Some classifications were associated with age (Moffitt “tumor”), pathological type (Moffitt “tumor”), pathological tumor size (Moffitt “stroma”, Collisson, Bailey), whereas all were associated with pathological grade, an important prognostic feature of PDACs. Based on the glandular cell differentiation, mitotic index, and nuclear atypia [13], the grade directly reflects important molecular characteristics of tumors, likely explaining its association with all these molecular classifications.

Next, we assessed the concordance of the four classifications in term of assignment to the poor-prognosis and good-prognosis groups in the 601-sample series. We combined the Bailey’s pancreatic progenitor, immunogenic and ADEX subtypes into a single good-prognosis group because their survival curves were not different. Similarly, we combined the Collisson’s classical and exocrine-like subtypes into a single good-prognosis group. In the Moffitt’s “tumor classification”, the “basal-like” subtype represented the poor-prognosis group, as did respectively the “activated” subtype in the “stroma classification”. Next, we compared the results of the classifications by using two-way contingency-table analyses. All comparisons showed significant correlations. The concordance rate (Additional file 5) was high (from 73 to 86%) between the three classifications based on gene lists derived from tumor tissues, and decreased when considering the concordance with the Moffitt’s “stroma classification”. Analysis based on Cramer’s V statistic (Fig. 2b) showed that the relation was strong between the Bailey and Moffitt’s “tumor” classifications and the Bailey’s and Collisson’s classifications, substantial between the Moffitt’s “tumor” and Collisson’s classifications, and low between each “tumor” classification (Bailey, Moffitt’s “tumor”, and Collisson) and the Moffitt’s “stroma classification”. With regard to the Cramer’s V values, the models showing the best and the worst agreements with the other ones were the Bailey’s classification and the Moffitt’s “stroma classification”, respectively. Thus, despite this little gene overlap, three of the four gene lists tested showed significant agreement in the outcome predictions for individual patients, probably tracking a common set of biologic phenotypes likely in part related to pathological grade. Of note, such high concordance further validated the robustness and coherence of all tumor classifiers.

Finally, we compared the prognostic value of all classifications. The 2-years OS in the Bailey’s classification were 23%, 48%, 56% and 46% in the squamous, pancreatic progenitor, immunogenic, and *ADEX* subtypes respectively (*p* = 5.78E-08, Fig. 1c). In multivariate analysis, the Bailey’s classification remained significant (*p* = 1.69E-02, Table 1). The 2-years OS in the Collison’s classification were 25%, 44%, 45% for the quasi-mesenchymal, exocrine-like, classical subtypes, respectively (*p* = 1.65E-03, Fig. 1d), but this classification lost its prognostic value in multivariate analysis (Table 1), as previously reported [7]. The comparison of the three other multivariate analyses including the clinical variables together with a molecular classification (Moffitt “tumor”, Moffitt “stroma”, Bailey) showed the most significant *p*-value with the Moffitt’s “tumor classification”. But multivariate analysis incorporating the four classifiers retained as significant the Moffitt’s “stroma” and Bailey’s classifications, suggesting independent prognostic value (Table 1). Similar results were observed in uni- and multivariate analyses when the 27 Collisson’s microdissected samples were excluded from analyses (data not shown), suggesting no impact of microdissection on our results.

## Conclusion

This prognostic analysis of molecular subtypes in PDAC is, to our knowledge, the largest series reported to date and the first study comparing these four promising classifications. We confirmed for the first time the independent prognostic value of the Moffitt’s classifications, and confirmed that of the Bailey’s classification, but did not confirm that of the Collisson’s classification. The gene overlap between all classifiers was low; there were many more common genes between the Collisson’s and Moffitt’s “tumor” gene lists and the Bailey’s gene list, derived in part or in totality from tumor cells, than between each of them and the Moffitt’s “stroma” gene list, derived from stromal genes only. Despite this little overlap, all classifications were associated with pathological grade. The concordance in term of outcome predictions was relatively high (from 73 to 86%) between the three classifications based on gene lists derived from tumor tissues, and low when considering the concordance with the Moffitt’s stroma classification. Despite higher prognostic value for the Moffitt’s “tumor classification” taken alone, the multivariate analysis incorporating the four classifiers together retained as independent variables the Moffitt’s “stroma” and Bailey’s classifications, highlighting the complementarity of classifiers based on tumor epithelium and stroma.

Our study displays some limitations related to the retrospective nature of data sets and associated biases, including the absence of information with respect to survival for all samples, However, our results reinforce the clinical validity of subtypes in PDAC. Of course, their clinical utility [14], or ability to improve patients’ management and outcome, remains to be demonstrated in prospective clinical trials, The clinical potential of subtyping is important. It provides new insights into the molecular pathophysiology of pancreatic cancer which may be used to tailor therapies. Many phase III clinical trials have failed to show benefit of tested agents in unselected patients with advanced-stage pancreatic cancer, although benefit was observed in occasional patients who may represent a given subtype in which they are selectively effective. For example, Collisson et al. defined preclinical models of their three subtypes and showed that gemcitabine and erlotinib were preferentially active in different subtypes [4]. Subtyping may also provide prognostic support in a clinical setting where the choice and timing of therapies is critical. For example, in early-stage disease, the subtypes could help select patients with resectable disease for either immediate surgery (for the good-prognosis subtypes) or neoadjuvant chemotherapy (for the poor-prognosis subtypes), which ultimately should affect outcome and impact quality of life. But of course, the clinical utility of subtyping remains to be prospectively demonstrated before any use in clinical routine. But yet, from a conceptual point of view the strong biological and prognostic differences observed yet suggest that PDAC should be regarded as a collection of separate diseases, providing a more homogeneous and favorable environment for identifying new prognostic and/or therapeutic targets and testing new therapies. Three take-home messages derive from our results. First, we need to identify, probably from the Moffitt’s “stroma” and Bailey’s classifiers given their complementary prognostic value, new biomarkers and/or therapeutic targets, which will reinforce the clinical interest of subtypes. Potential therapeutic targets include for example immune modulators such as checkpoints inhibitors in the Bailey’s immunogenic subtype, drugs “normalizing” the TP53, TP63 and KDM6A pathways frequently altered in the Bailey’s squamous subtype, or drugs targeting PDAC stromal components, notably the pancreatic stellate cells or specific fibroblast subsets [15], in the Moffitt’s “stroma” subtype. Second, PDAC subtypes are predictive of the prognosis, with the mesenchymal subtype being the worst of all, as already reported in breast cancer with the basal subtype [3]. Third, the pancreatic tumor microenvironment contributes to the prognosis, and adds prognostic information to classifiers based on “tumor epithelium” genes.

## Additional files

Additional file 1: Table S1. List of pancreatic cancer data sets included (XLSX 10 kb)

Additional file 2: Table S2. Clinicopathological characteristics of samples and correlations with molecular subtypes of the four classifications (XLSX 14 kb)

Additional file 3: Table S3. Gene overlapping between the four classifiers (XLSX 8 kb)

Additional file 4: Table S4. Common genes between the four multigene classifiers (XLSX 14 kb)

Additional file 5: Table S5. Concordance rate between all classifications compared two-by-two in term of outcome prediction (good- or poor-prognosis). All comparisons were significant by using two-way contingency-table analyses (Chi2-test) (XLSX 8 kb)

## Abbreviations

ADEX: aberrantly differentiated endocrine exocrine
AJJCC: American Joint Committee on Cancer
OS: overall survival
PDAC: pancreatic ductal adenocarcinoma
